# Reports of Cases Occurring in “Bellevue Hospital,” Richmond, Virginia

**Published:** 1860-04

**Authors:** Thomas Pollard

**Affiliations:** One of the Physicians


### ARTICLE II.

*Reports of Cases occurring in "Bellevue Hospital," Richmond, Virginia.* By THOMAS POLLARD, M. D., one of the Physicians.

MR. EDITOR—The following cases selected from our Hospital records, for publication, are presented not so much for their novelty, or success of treatment, as for the opportunity several of them afforded of verifying the diagnosis, and prognosis made during life, and as contributions to pathological anatomy. While "autopsies" are not flattering to the professional pride of the medical man, they are always instructive, and at least have the merit of something tangible and definite—a merit wanting to many reported remarkable and astonishing "cases."

CASE 1—*Chronic Cystitis.*—Seymour, a slave, aged 45, admitted Nov. 8, 1859. General health bad, having been the subject of "Cystitis" for some length of time. On examination with the catheter, and with the finger per rectum, the prostate gland is found considerably enlarged—dysuria very decided, and a catheter has to be introduced three times a day to draw off the urine. Diarrhœa is another troublesome symptom, and this with disease of bladder has rendered him pale, emaciated and feeble.

℞.—Six leeches to Perineum; submur. hyd. grs., v; pulv. dov. grs., viij., at bed time, with warm hip bath.

November 9.—On examining urine, found it to contain great mucus deposit: slept badly.

℞.—Infus. buchu ℥ ij., every 3 hours until bed time; then pulv. dov. without the calomel.

Nov. 12.—Condition more unfavorable; rigors, with complete retention of urine, which is drawn off by the catheter.

℞.—Apply a blister over the region of the bladder, to stay on six hours. Diarrhœa still troublesome, for which bismuth has been used.

Nov. 14.—Pulse found to be growing weaker on yesterday, and milk toddy was ordered—continued to sink, and died to-day at 3, P. M.

Autopsy 12 hours after death—Prostate found greatly enlarged, with constriction of prostatic portion of the urethra The walls of the bladder were *six* or *eight* times as thick as usual, and the mucus membrane was in a softened, pulpy condition, showing disease of long standing. Kidneys very much enlarged and softened, breaking down under the finger. Liver softened. Mucus membrane of the intestinal canal injected, and softened. The liver also softened, and in some places nearly disorganized. The enlargement of the prostate gland, was probably in this case, the starting point. To overcome the resistance offered to the expulsion of the urine, (by the enlarged gland,) nature caused hypertrophy to be produced in the walls of the bladder as a compensating power. The disorder and softening of the other organs were probably secondary effects of impaired general vitality.

CASE 2—Death from Chloroform. Albert, a slave, aged 30, admitted Jan. 9, 1860, was placed upon the bed to undergo the operation for radical cure of Hernia as proposed by Wutzer. Finding that the resistance made by the patient was so great as to prevent the performance of the operation, the Surgeon directed Chloroform to be administered only to *partial anesthesia.* About two ʒ 's of chloroform were poured upon the sponge, and administered in the usual way, admitting a free access of atmospheric air. The agent had been administered about one minute, when a severe convulsion occurred, and with it, death.

Autopsy nineteen hours after death—Great congestion of the brain was found, the sinuses being particularly distended with blood. The lungs were congested, especially at the base, which may have been due partly to position. The heart was healthy.

The man had previously been in good health. The immediate cause of death in this case is uncertain. The residuum of air left in the lungs was probably too small at the time of the administration of chloroform, for the patient had been actively struggling to escape the operation, and was much frightened. The suddenness of the death, occurring in the midst of a general convulsion, would seem to point to spasm of the epiglottis, cutting off a supply of air to the lungs.—This possibly might have been borne for a short time, if the residuum of air had been good. The congestion of the brain was, perhaps, the cause of the convulsion, and may thus have been the first link in the chain of morbid action. This is the first death which has been caused by the use of chloroform in this Hospital, and, as far has been made public, the first which has resulted from the use of this agent in this city, though it has been freely administered. There is some reason to believe the chloroform used in this case, was not pure, though it has not yet been subjected to chemical examination. Its appearance (not being entirely clear and pellucid) is the ground of suspicion that the agent might not have been pure.

This result of the use of Chloroform, is another argument in favor of Ether, which is now being so much substituted for chloroform.

CASE 3.—Successful operation for "Radical cure of Hernia." French, a slave, aged 10, admitted Nov. 15, 1859, was operated on, on the 19th, by Wutzer's method, modified by Rothmund. Chloroform was not used. The instrument was removed on the 2d December, and a compress applied. The inflammation was not great. The intestine remained up for a time, but ultimately came down. The operation was repeated on the 16th Dec., and the patient was discharged on the 22d of February, 1860, sixty-eight days after the operation, apparently cured. The parts were supported by Chase's truss before dismissed, as a safeguard against a descent of the intestine. The hernia in this instance said to be congenital. This operation thus far is not considered a very successful one; and it is said that the European surgeons have given it up. Apparent cures certainly are effected, and the American surgeons are yet persevering in the operation.

CASE 4.—Chronic Laryngitis. Mary, aged 25, black, was admitted Dec. 7, 1859, with great hoarseness of voice, occasional cough, and much dyspnea. On looking into the throat the epiglotis was discovered much swollen, and prominent. The lungs seemed in a healthy condition, except that the due quantity of air was not admitted.

℞.—Nit. argent, ʒj., to water, ℥j., to be applied by probang to throat, and top of the larynx once a day; croton oil to be rubbed externally over the larynx, to pustulation; cod-liver oil, ℥ss., with 5 grs. iod. potass., three times a day.

In the application of the nit. argent, the endeavor was to apply the mop on the top of the epiglottis, and permit the solution to trickle into the larynx. Under this treatment the patient gradually improved. The voice was strengthened, the dyspnea diminished, and the general condition of the patient was much better. On the 2d of February, 1860, she was so much improved that her master determined to remove her. She had not gone a hundred yards from the hospital in a comfortable carriage, when she suddenly expired, and her master speedily returned with her. On the day of her death, as on many previous days, she was freely walking about the hospital, and rarely laid down.

Autopsy 20 hours after death—The epiglottis very much thickened and enlarged, and having the appearance of fatty degeneration ; in fact the whole larynx partook of the same appearance. The mucus membrane was thickened and injected, particularly in the upper part of the trachea, and in the lower part of the larynx was some ulceration. The lungs were healthy, with the exception of some congestion. It seemed almost a miracle that the larynx and epiglottis performed their function as long as they did, with so much disease.

The immediate cause of death in this case was, no doubt, spasm of the glottis, brought about by the sudden impress of cold. The day she died, the 2d of February, was extremely cold, and the atmosphere from which she was carried, was very warm.

CASE 5.—Mumps terminating fatally. Abraham, black, aged 25, entered Jan. 19, 1860. The parotid gland on each side was swollen. A laxative of castor-oil was ordered, and a stimulating liniment to the glands.

Jan. 21.—Swelling almost entirely subsided. The patient was examined by the attending physician, about 12 o'clock, and nothing unnatural discovered about him. He was walking about the room, and seemed quite well—had eaten his usual breakfast, and his bowels were in good condition.—There seemed no indication for any treatment. About 1 o'clock, I rode up to the Hospital, and saw Abraham come rapidly down the steps and run off. Supposing he had "run away," I despatched two servants after him immediately.—They overtook him after he had run 150 yards, and leaped a high pailing. After walking half the distance back, he suddenly sunk to the ground, and had to be conveyed by several assistants, to his room. On feeling his pulse it was found to be very flickering and feeble, and in spite of a large draught of brandy which was speedily given him, he rapidly sunk, and in ten minutes was dead.

It was afterwards ascertained he had been a drinking man, but his master thought he had stopped drinking for the last 12 months, and he had not the appearance of a drunkard.

The cause of death is obscure in this case. There may have been some metastatis to the brain, but there was no evidence of it, unless the sudden mental disturbance, which caused him to run off, was an indication of it. The greater probability is, that he had been on the verge of "Delirium Tremens" for a day or two, and that it suddenly manifested itself, and caused the desire to escape. Death must have resulted immediately from exhaustion, asthenia. His exertion had been violent, and the action of the heart was so far depressed as to prevent its reaction. No *post mortem* was allowed.

CASE 6.—Phthisis—use of Lead. Martha, aged 35, entered July 16, 1859. A cavity was found to exist in the top of both lungs. She was placed on the usual tonic and supporting treatment, with cod-liver oil. There was improvement in the symptoms.

September 10.—She was put on the "carbonas plumbi" as recommended by M. Beau, at the same time that the cod-liver oil, and other treatment was continued. The dose in the beginning was 2 grs. per day, the dose being gradually increased to 12 grs. per day. The improvement progressed for a little while, but not of long continuance, or decided in character. No sign was perceived of the constitutional effect of the lead, nor was the blue line manifested on the gums, though the medicine was kept up until Nov. 9.

Death took place on Nov. 5. *Post-mortem* examination showed large cavities in each lung, with extensive tubercular deposits. The intestines were rendered blue by the lead, throughout the whole extent, and the muscles both of the upper and lower extremities, as well as those of the abdomen, were almost perfectly blue.

CASE 7.—Phthisis—Lead Treatment. James, black, aged 40, entered September 19, 1859. Examination of the lungs revealed consolidation of the apex of both of them from tubercular deposit, and proionged *expiration* was manifested over the whole chest. The expectoration was bloody, and sometimes pure blood in considerable quantities. The cough was severe, the pulse accelerated, and night sweats were of frequent occurrence. The patient was placed upon cod-liver oil, and 2 grs. carbonate lead, three times a day, and the dose gradually raised to 12 grs. per day. In about fifteen days the blue margin appeared around the gums; the symptoms in the mean time progressively undergoing improvement, and the general condition also much improved. The constitutional effect of the lead being obtained, the dose was reduced to 3 grs. daily, which kept up the effect already attained. No colic, or other unpleasant effects resulted from the lead. On the 17th November the patient was discharged apparently almost well. The cough was nearly, or quite gone; the sweats had disappeared; the breathing was nearly natural, and percussion evinced little or no dullness where it had been most distinct before. The man looked hearty and said he felt well.

The patient has recently presented himself at the hospital, and seems cured; and he says he is as well as he ever was in his life.

This case cannot be fairly brought forward as an evidence of the success of the lead treatment, because other remedies were used, but certain it is that the man had all the evidences as phthisis precedent to the formation of a cavity, and that he is now apparently well. The part the lead bore in the treatment cannot be certainly stated, and its value can only be decided by future observations.

CASE 8.—Cancer of the Æsophagus. Biddy aged 40, was admitted Dec. 10, 1859. Her symptoms were regurgitation of food, great difficulty of swallowing, even water, and emaciation and loss of strength. These symptoms had existed in less degree for several months. Recently they had become more urgent. Scarcely any fluid was taken which was not returned—the patient had ceased to attempt the deglutition of solids. An attempt was made to introduce the tube of a stomach-pump. It passed a small stricture half way down the æsophagus, and stopped obstinately two-thirds of the way down. A gum-elastic probang was then used, but with no better success, and some gutta-percha bougies manufactured for the purpose, of small size, also refused to pass. No instrument, indeed, could be gotton to the stomach.—Palliative treatment with nourishing enemata, and occasionally a purgative enema, were the only things resorted to—emaciation progressed, and the strength rapidly failed, and death took place on the 27th December.

P. M., 12 hours after death.—A ring half way round the æsophagus was found about the middle of the tube. This was hard, and of scirrhus appearance and feel. Two-thirds of the way down the principal stricture was found. This was hard and dense, and unyielding. A small bougie only could be passed, and this through a tortuous passage in the æsophagus; and the relaxation of death permitted the passage of an instrument, which during life could not be made to pass. Probably some fluid nourishment percolated through this narrowed channel, and thus supported life for some time. This larger deposit producing the lower stricture was undoubtedly schirrhus. Some ulceration, it should have been stated, was found in this lower stricture, and the patient had during life been discharging some offensive, sanious pus.—The stricture higher up was additional proof, if any were wanting, of the constitutional character of the malady.

